# On the Typhus Fever Which Occurred at Philadelphia in the Spring and Summer of 1836; Illustrated by Clinical Observations at the Philadelphia Hospital; Showing the Distinction between This Form of Disease and Dothinenteritis or the Typhoid Fever with Alteration of the Follicles of the Small Intestine

**Published:** 1841-10

**Authors:** 


### BRITISH AND FOREIGN
### MEDICAL REVIEW,

FOR OCTOBER, 1841.

### PART FIRST.

### Analytical and Critical Reviews.

### Art. I.

1. *On the Typhus Fever which occurred at Philadelphia in the spring and summer of* 1836; *illustrated by Clinical Observations at the Philadelphia Hospital; showing the distinction between this form of disease and Dothinenteritis or the Typhoid Fever with alteration of the follicles of the small intestine.* By W. W. Gerhard, M.D., one of the Physicians to the Hospital. (*American Journal of the Medical Sciences.* Nos. 38 and 40, February and August, 1837.)

2. *Considérations sur la Fièvre Typhoide, &c. &c.—Du Typhus Fever et de la Fièvre Typhoide d'Angleterre.* Par M. Valleix, M.D. (*Archives Générales de Méd.* Janv. Fevr. Octob. et Novemb.)—*Paris,* 1839. 8vo.

*On the Typhoid and Typhus Fever of France and England.* By M. Valleix, M.D.—*Paris,* 1839.

3. *Report on the Epidemic Fever of Edinburgh. An Account of the Symptoms and Treatment.* By William Henderson, F.R.C.PH. &c.

*Analysis and Details of Forty-seven Inspections after Death.* By John Reid, F.R.C.PH., &c. (*Edinburgh Medical and Surgical Journal,* Vol. LII. pp. 429-462.—*Edin.* 1839.

4. *Mémoire sur le Fièvre Typhoide, sur les diverses Formes qu'elle peut présenter, et sur le Traitement qui lui est applicable,* (*Mémoire honoré d'une médaille d'or par la Société Médicale de Toulouse.*) Par J. B. Delaroque, Médecin de l'Hôpital Necker, &c.—*Paris,* 1839. 8vo, pp. 235.

*Essay on Typhoid Fever, its different Forms and mode of Treatment, &c.* By J. B. Delaroque, Physician to the Necker Hospital.—*Paris,* 1839.

5. *Der Abdominal Typhus.* Monographische Skizze von Dr. F. Cramer.—*Cassel,* 1840. 8vo, pp. 128.

*On Abdominal Typhus.* By F. Cramer, M.D.—*Cassel,* 1840.

6. *The Articles "General Doctrines of Fever" and "Continued Fever."* By Dr. Christison, (*Library of Medicine,* vol. I. pp. 113-188.)—*London,* 1840.

7. *Lectures on the Morbid Anatomy of the Serous and Mucous Membranes.* By Thomas Hodgkin, m.d. (Vol. II. Lectures xxii. and xxiii.)—*London*, 1840.

8. *Some Considerations on the Nature and Pathology of Typhus and Typhoid Fever, applied to the solution of the question of the Identity or Non-identity of the Two Diseases.* By A. P. Stewart, m.d. (Read before the Parisian Medical Society on the 16th and 23d of April, 1840.)—*Edinburgh Medical and Surgical Journal*, Vol. LIV., pp. 289-339. October, 1840.

9. *Observations on Typhus, submitted to the Faculty of Physicians and Surgeons of Glasgow.* By Andrew Anderson, m.d. &c.—*Glasgow*, 1840. 8vo, pp. 66.

10. *Traité de l'Entérite Folliculeuse* (*Fièvre Typhoide*). Par C. P. Forget, Professeur de Clinique Médicale de la Faculté de Strasbourg, &c. &c.—*Paris*, 1841. 8vo, pp. 856.

*Treatise on Follicular Enteritis* (*Typhoid Fever*). By C. P. Forget, m.d., Professor of Clinical Medicine to the Faculty of Strasburg, &c. &c.—*Paris*, 1841.

11. *Recherches Anatomiques, Pathologiques et Thèrapeutiques sur la Maladie connue sous les noms de Fièvre typhoïde, putride, adynamique, ataxique, bilieuse, muqueuse, gastro-entérite, entérite folliculeuse, dothinentérie, &c., comparée avec les maladies aigues les plus ordinaires.* Par P. C. A. Louis, m.d., Médecin de l'Hôtel-Dieu, Président perpétuel de la Société Médicale d'Observation, &c.—*Paris*, 1841. 2 tomes, 8vo, pp. 542, 523.

*Anatomical, Pathological, and Therapeutical Researches on the Disease known under the names of Typhoid Fever, &c.* By P. C. A. Louis, Physician to the Hôtel-Dieu, Perpetual President of the Medical Society of Observation, &c.—*Paris*, 1841.

In our last Number we presented the readers of this Journal with a tolerably close analysis of the volumes of M. Louis upon Continued Fever, and in the course of this announced our intention of pursuing the subject, and more especially of examining the much-litigated question of the identity or non-identity of the disease, as it exists in France and in our own country. Accordingly we proceed to examine with as much fulness as we may reasonably venture on, the state of facts and opinions, as displayed in some of the most recent works published on both sides of the channel. We shall endeavour to render the basis of comparison still more complete by a brief enquiry into the history of the malady observed in other continental countries, and in the United States. Having submitted, as we pass, the work of each author to the criticism apparently warranted by its pretensions, we shall draw our conclusions from the whole mass of evidence brought before the reader.

One of the authors whose names appear at the head of this article, an enthusiastic advocate of the doctrine ascribing to the lesion of Peyer's glands all the phenomena of typhoid fever, commences his bulky tome with an elaborate enquiry into the opinions respecting the nature and essence of the malady entertained by our forefathers. We shall not accompany the writer, M. Forget, seriatim through the details wherefrom he considers himself justified in concluding that all historical evidence goes to prove the acknowledgment from the time of Hippocrates to the present day of three fundamental propositions in the doctrine of fever: namely, the identity of the different forms of continued fever; their inflammatory character; their seat in the intestine. Our limited space prevents us from undertaking this task in its full extent; but it will not be beyond our means, nor irrelevant to the purpose of the present enquiry, to take a rapid survey of the more important passages adduced by this ingenious ransacker of bygone literature, as susceptible of the interpretation he has found for the febrile doctrine of antiquity. It must, in justice to M. Forget, be admitted that his quotations are correct, and if it be fair to build upon particular passages general doctrines, to argue from the literal acceptation of a phrase, while the *animus* of its author throughout the pages in which it occurs is wholly lost sight of, M. Forget may claim the honour due to a faithful historical investigator. It is true that Hippocrates has said "febres vertiginosæ, et cum tenuis intestini morbo, et sine hoc, perniciem intentant," but, as even M. Forget shrinks from regarding these words as more than the accidental expression of a notion vaguely conceived and enunciated without conviction, it is needless for us to set about proving what is self-evident. Baillou, no doubt, asserts his belief that "almost all fevers (with the exception of some symptomatic ones) have their cause in the mesentery," and declares that "the surface of the intestines is inflamed;"* but these opinions are far from pervading his works. Willis notes the frequency of dysentery, as he terms it, during continued fever, speaks of the formation of small ulcers in the intestine, and compares this internal pustulation to the external of variola, a parallel for the honour of originating which, superficial and fallacious as it is, contemporary French authors have contended. The passages wherein the illusory nature of common belief respecting the "malignity" of fever is vigorously stigmatised by Sydenham, are probably too well known by English readers to require particular citation. Sydenham did not recognize any connexion between continued fever and intestinal lesion, but in ascribing "malignity" to intense inflammation he appears to M. Forget to lend aid—truly, most garbled aid—to the establishment of the thesis we have stated. Chirac observed, as he alleges, redness, inflammation, and livid spots in the stomach and intestines of subjects dying of fever "before the fourth day." Baglivi professes notions of the same stamp as Baillou. Frederic Hoffman states that "putrid synochus, ardent, bilious, inflammatory fever, are accompanied with inflammation of the stomach and intestines." Huxham speaks of certain symptoms as "generally originating in inflammation or mortification of the intestines." If Pringle's theory ascribed "the continuation of fever to early inflammation of some part of the brain or nervous system," he also speaks of the intestines as being "more particularly subject to inflammations." Roederer and Wagler, in their celebrated work *De Morbo Mucoso*, carefully describe the condition of Peyer's patches, and ascribe the fatal result in fever to abdominal inflammation and gangrene or to engorgement of the lungs. Sarcone, ob-

* De Urinar. Hypostasi.

serving at Naples, maintained the alimentary canal and the entire abdominal system to be the parts, next to the blood, most constantly affected; his countryman, the celebrated Cotugno, observed enlargement of the mesenteric glands. Cullen is appealed to by M. Forget as upholding the doctrine of the identity of all "essential fevers," though his nosographical arrangements tended to keep alive the idea that his notions were of a very different kind. Thus he speaks of synochus and typhus as being produced by the same causes, and therefore varieties of each other; looks upon typhus as a genus comprehending several species, a great number of which acknowledge no specific difference, and appear to be merely varieties depending upon accidental circumstances. Stoll's testimony to the existence of gastro-intestinal lesion in fever is distinct and unquestionable; but he regards this as the result of irritation induced by saburral matter circulating in the bowel. The doctrine of essentiality taught by Pinel, and his division of continued fever into species, is denounced by M. Forget as having thrown the science back. Now we mean not to defend either branch of this doctrine, for we believe both to have been totally erroneous; but we deny that their promulgation changed the prospects of the science in the least, for the simple reason that Cullen and others of the authors cited by M. Forget preached a theory fundamentally the same. But M. Forget evidently knows something more of Pinel than he does of Cullen. Pass we next to one of the most zealous labourers, produced by the positive tendencies of the age, in the discovery of the organic cause of disease—Prost, the real originator of the gastro-enteritic theory of fever. Prost wrote in 1804: "I had made at least 150 examinations of the bodies of persons dying of ataxic fever, without being able to discover anything particular in the brain; but I had *always* seen inflammation of the intestinal mucous membrane with or without excoriation. . . . . Mucous, gastric, ataxic, adynamic fevers have their seat in the mucous membrane of the intestines; they result from divers morbid changes in that membrane."* In 1813 Petit and Serres described their "entero-mesenteric fever," defined with tolerable clearness the lesions of the patches of Peyer and mesenteric glands, distinguishing them from common inflammation; they taught that this morbid condition precedes the development of fever, and that the danger of this latter is always proportional to the degree of the former. The year 1816 was marked by the advent of Broussaisism. How completely that doctrine was anticipated, as regards its founder, is manifest from the passage just quoted from Prost. Prost sank to the grave unhonoured and unthought of; Broussais was almost deified at the very outset of his career: the cause is plain; the latter cried aloud till he was heard, the former contented himself with a modest statement of what he believed himself to have observed; the one wrote with the energy of conviction, the other with the feebleness of indifference. The result exhibited once more the triumph of *manner*, realizing even in matters of science the saying of Chateaubriand: "*C'est par le style qu'on vit.*"

There cannot, we imagine, exist a particle of doubt in the mind of any one acquainted with the general doctrines of the authors referred to, that those doctrines are grievously misinterpreted by M. Forget. Indeed,

* Médecine éclairée par l'ouverture des corps, t. i. p. viii.

to English readers the perversion must be so apparent, that for their sakes we should not have considered it necessary to proclaim it; but as in France the historical manifesto of the Strasburg Professor is likely to be triumphantly appealed to by the Broussaisian school, it is incumbent upon us to enter a protest against its justness. We have no inclination to contest M. Forget's right to close his volume with the "*Cecy est un livre de bonne foy*" of Montaigne; but, if so, it is clear that with *bonne foy* alone a man cannot write history. However, the truth is, that what the general theories held by these departed worthies may have been, is, comparatively speaking, matter of little moment: the important point is to determine what the just inference is from the facts they observed and registered; the history of things, not of opinions. Now an examination of the entire of these works, especially the English, and exclusive of all those produced from the time of Prost downwards, shows that the true version of their authors' experience in respect of M. Forget's three propositions is this: continued fever is sometimes attended with disease of the intestine; fever is commonly attended with, if it do not necessarily involve the presence of, inflammation; the different forms of continued fever are all probably referrible to a single species. These notions are very different from those putatively gleaned from antiquity by M. Forget, and condensed by him into the terse dogmata—seat in the intestines, inflammatory nature, identity; but they are precisely such positions as the latitudinarian interpretation of a hot and ingenious partisan might most easily convert into those put forth by this writer.

The opuscule of M. Delaroque, although "got up in an extreme hurry" for presentation to the Medical Society of Toulouse, will, the author dares hope, satisfy the desires of "practitioners." We feel abundant gratification in being able to reassure one suffering under such modest misgivings as to the perfection of his doings: it will more than satisfy them, it will enrapture them, if by "practitioners" we understand, with M. Delaroque, that host of "good easy men," who ask neither for logic nor philosophy, but cry aloud for a drug for each malady, and fancy themselves practitioners, when some culler of simples answers *eccola!* To these persons M. Delaroque's pages will be an invaluable gift, pages announcing in purgatives a panacea for typhoid fever. Whether drastics deserve this character or not, however, it is not our purpose to enquire; and had the author contented himself with developing such asseveration, his labours would not have been brought under notice in the present article. But no, the alleged fact is not enough, a theory of the disease must be found; M. Delaroque having worked marvels in therapeutics, covets the fame of a pathologist. Accordingly we are taught that typhoid fever is the mere "product of the action of decomposed fluids on the alimentary canal," which are presumed, having first stagnated at the end of the ileum, to enter the circulation. Here the organic origin of the disease is set aside, and the saburral doctrine of Stoll restored; a doctrine "dating from so early a period," (what will the historic penetration of M. Forget say to this?) "that it is to be found in Hippocrates, and in all the authors of antiquity." Besides, adds M. Delaroque, "it is the only theory accordant with the daily results of treatment." The only theory accordant with the results of *my* treatment—*hinc illæ lachrymæ;* we suspect, in truth, the sweet voice of antiquity might have sung in vain, had not M. Delaroque gained a certain Parisian notoriety by treating typhoid fever with purgatives in the morning, purgatives at noon, purgatives in the evening, and purgatives in the morning again.

Possessed of a very superficial acquaintance with the malady he describes,* M. Delaroque unhesitatingly gives forth the law upon every possible point, and sneers at those who have profoundly laboured in its investigation. All this is the natural, and almost the necessary, consequence of the attempt to found a theory of a disease upon presumed results of treatment. Compare M. Bouillaud, who affirms that the disease *must* be a violent inflammation, because he *cures* it by extracting almost all the blood in the body: M. Delaroque, who is equally positive the whole malady *must* consist in the presence of saburræ, because by submitting the small intestines to a process of perpetual scouring he *cures* the disease: and lastly M. Piedagnel, who (if he were to follow in the same strain) has stronger reasons than either for declaring the malady *must* be no malady at all, inasmuch as he *cures* more patients than any one by doing nothing at all.†

Let us now return to the volumes of Louis, the main doctrines of which may be enunciated as follows: 1. The different species of continued fever admitted by Pinel are all referrible to a single malady, the typhoid affection. 2. Their anatomical character is a special lesion of the patches of Peyer, and of the mesenteric glands, a lesion inseparable from its existence, and one which developes itself in a more regular and fixed manner than the anatomical character of any of the phlegmasiæ. 3. This lesion is developed before any one of the other organic changes that attend the disease, except probably an altered condition of the blood. 4. It is specific, differing in its characters from those of ordinary inflammation. 5. The general tract of the gastro-intestinal surface is not necessarily implicated. 6. The disease is attended with marked proneness to membranous ulceration. 7. The typhoid affection is not strictly assimilable to the exanthemata. 8. It occurs but once in a lifetime. 9. It is not observed in individuals who have passed a certain age, about fifty. 10. It originates spontaneously and in contagion.

The first point we have to enquire into is, whether the doctrines here maintained are borne out by general Parisian experience. Respecting the fundamental identity of the different species of "essential fever" established by Pinel (and these answer to the different forms of continued fever with or without organic complication admitted in this country), no doubt is now hazarded by any of the more observant physicians of Paris. M. Chomel, M. Bouillaud, and all others who have given public expression to their experience, agree in regarding the class of essential fevers as a single malady, varying accidentally in its symp-

* M. Delaroque leaves acquaintance with the anatomical history of the disease to such persons as MM. Louis, Andral, and Chomel, "*who have occasion to make many post-mortem examinations.*" He, of course, can have no such opportunity; live but the purgative system awhile, and the dissection of a patient with typhoid fever will be matter of tradition.

† Of sixty cases allowed by M. Piedagnel to run their own course, three only terminated fatally, the lowest ratio of mortality ever obtained in Paris.

tomatic manifestations, but under all circumstances equally characterized by the existence of the glandular lesion of the small intestine, and capable of being referred thereto during life in spite of any degree of variableness of those manifestations ever actually witnessed.

Some discrepancy of opinion prevails, as has already appeared in our former article (No. XXIII. p. 22), regarding the invariable and necessary presence of the special lesion of Peyer's glands in subjects labouring under the continued fever of Paris. The argument of M. Dalmas, and the species of refutation adopted by M. Valleix, have already been mentioned. It is not easy to detect sophistry in the reasoning of the latter; it is difficult even to assign just cause for not adhering to it altogether, yet it has been by some esteemed rather more ingenious than solid, and that an *arrière-doute* leaves the mind unsatisfied by its perusal cannot be denied. And this doubt is strengthened by the fact, that Andral concludes from his experience, that "in the pyrexiæ constituting the different morbid groups designated by the title of essential fevers, certain lesions are very frequently, ninety-eight times in every hundred about," [but only this] "detected in the digestive tube;" he believes, however, that "when continued fever is connected with the intestinal exanthema, the latter originates at the outset of the disease." M. Chomel, as previously mentioned, has not encountered a single case of typhoid fever within the last five years unattended with the Peyerian lesion, yet in respect of general assertion, he conceives himself warranted in going no further than the affirmation, that "if it be not constant in the strict acceptation of the term, its total absence is extremely rare." M. Rostan has within the last year taught that continued fever does in rare instances exist in Paris without the Peyerian disease.

A serious objection of the converse kind has been raised to the doctrine; it has been affirmed, that lesions undistinguishable from those developed as attendants on typhoid fever, occur in other diseases; among acute, in scarlatina, epidemic cholera, and yellow fever; among chronic, in phthisis. Further, the intestinal patches of infants are said frequently to present a similar state of change without (the presumption is) symptoms of typhoid fever having preceded death. That under some of these circumstances the patches swerve from the normal state is admitted by the majority of observers; but, as we can affirm from experience, confusion can only arise in a certain number of them, if the examination be conducted with extreme inattention. Thus, in scarlatina, redness and thickening of the patches is occasionally observed, but such state has not been known to lapse into ulceration: M. Forget suggests that in such cases the symptoms during life may have worn an ataxic or typhoid character. In the majority of the victims of cholera observed by M. Chomel, the follicles of the intestine were enlarged; but the lesion differed from that of continued fever, in that the elevation of the patches was much less considerable, that the change was of uniform character at all periods of the disease, from twenty-four hours to thirty-six days after seizure, and that ulceration was never detected. The minute enquiries of Böhm, though not precisely corroborating the description of M. Chomel, nevertheless bear him out in his affirmation, that a difference exists between the lesions developed in the two diseases. Böhm, whose previous researches particularly qualified him for such enquiry, describes two kinds of alteration in cholera, "one affecting the mucous, the other the submucous tissue. The mucous surface of the Peyerian glands is destroyed, the small capsules ulcerate, open, and disappear, even to their outermost border. The cavities of the capsules thereby become minute fossulæ, and the surface of the patches mesh-like or retiform. The submucous tunic is sometimes filled with and swollen by a white plastic exudation. Still this change never reaches the degree in cholera that it does in typhus abdominalis."* According to the researches of M. Louis, the glandular patches maintain the natural appearance in yellow fever. The objection drawn from the condition of the glands in early infancy, loses its force in a great measure from the circumstance that the absence of continued fever during life does not appear to have been established. The diseased patches in phthisis, instead of being infiltrated throughout their cellular substratum with whitish yellow matter, are studded with minute tubercles; and where extensive ulceration has occurred, the case may be still distinguished either by the presence of tubercles on the edge of the ulceration, or by thickening and induration of the surrounding tissues; besides, the mesenteric glands are infiltrated with tuberculous matter.

Respecting the initiatory character of the lesion, when it exists, we have shown by a quotation that M. Andral's experience agrees with that of M. Louis: the character of the earliest symptoms is the ground of such belief, coupled with the fact of its being confirmed by such dissections as have been performed almost at the outset of the malady; on the 8th, 7th, 6th, (Montault, Andral,) even 5th days, (Bretonneau, Forget,) the special morbid change has been discovered. But by M. Louis, at least, the primitive character of the lesion is only asserted as regards appreciable changes of tissue: that the blood is the real seat of the primary change was pronounced in his first edition (vol. ii. p. 311) "far from improbable, and indeed in many cases to be strongly presumed:" an opinion adhered to in the second (vol. ii. p. 178). M. Chomel professes the same belief: M. Rostan has espoused it.

We have stated the reasons which induced M. Louis to regard the inflammation of the mucous tissue of the patches as of specific, or if the term be objected to, of peculiar character. The main reason for this consists in the development of morbid matter in the submucous tissue, an occurrence which the researches of Böhm, and, we might add, those of M. Chomel, prove to be of even more frequent occurrence than the experience of M. Louis warranted him in affirming. The disciples of the gastro-enteritic theory, disciples whose ranks grow daily thinner, while the relics thereof adhere to it with all the petulant pertinacity of a defeated party, naturally look with an evil eye upon, and are perpetually carping at this doctrine. M. Forget scouts the notion of any peculiar character appertaining to the phenomena attending the development of the Peyerian lesions, and apostrophises the holders of the opposite opinion in language displaying the vivacity of the enthusiast, but neither the soundness of the logician nor the caution of the observer.

The correctness of M. Louis's inference respecting the secondary importance of lesion of the general surface of the gastro-intestinal mucous

* Muller's Archiv. Heft iv. 1839.

tunic is, as might be anticipated, also contested by the followers of Broussaisism. But when M. Bouillaud exclaims that "none but observers blinded by the spirit of prejudice . . . . can maintain that the inflammation resides exclusively and solely in the patches of Peyer," he alters the terms of the question. MM. Louis and Chomel, against whom the tirade is evidently directed, have not affirmed as a general proposition that the follicular structure is the sole seat of disease in the digestive tube; their facts would not warrant any such inference: but these facts do prove that the affection may terminate fatally in rare instances without material implication of the general mucous surface. M. Bouillaud's partisanship prevents him from going further than the admission, that "the principal disease exists in the patches and in the isolated follicles:" the truth, but not the whole truth.

The proneness to membranous ulceration displayed by the anatomical details of M. Louis, has been confirmed by others, though perhaps not quite to the same extent.

That the disease occurs but once in life; in other words, that a first attack ensures immunity from a second, is a point in its history respecting which opinions in Paris appear to be unanimous.

M. Louis has attached considerable diagnostic importance to the alleged fact, that typhoid fever is a disease of youth and of adult age; he himself never having observed it in subjects who had passed the fiftieth year. The perfect correctness of M. Louis' opinion may be doubted. In the first place the experience of others does not corroborate it; thus Petit observed the disease in a subject aged sixty, M. Montault in one of sixty-five, M. Andral in one aged seventy. In the second place M. Louis has observed at an hospital—and this is true of the other professors whose names have been repeatedly mentioned—into which sexagenarians rarely find their way; and we have not known it affirmed that in the immense establishments devoted to their reception, the development of the malady has been demonstated to be either unknown or extremely rare. There seems to be a strong reason of another kind for supposing that the occurrence of the disease must be exceedingly uncommon at an advanced age, at least among the individuals forming the ordinary population of hospitals, namely, the vast frequency among the youthful and adult of the lower orders of Paris, coupled with the ascertained fact that it is a malady that occurs once only in a lifetime. M. Louis, however, in anticipation of this objection, argues its want of validity on the ground that the immunity from a second produced by a first attack of variola, of measles or scarlatina, and the equal frequency of these diseases in infancy, are well established, "yet no age is exempt from the chance of variola, measles, or scarlatina." But to this it may be replied that, as the experience of M. Louis' colleagues proves, no age is exempt from the chance of typhoid fever.

The Parisian school generally has been resolutely anti-contagionist, founding its belief on the line of argument attributed in our last article to M. Andral. M. Chomel was perhaps the first to waver in his acceptance of the prevalent doctrine. He maintained that the fact of non-contagion in Paris had never been demonstrated, inclined to the belief that the malady was actually contagious to a limited degree, and suggested that this would become matter of certainty should subsequent researches prove the identity of the anatomical lesions of typhoid fever and camp typhus. We have exhibited M. Louis' arguments in favour of contagion prevailing in Paris, and we certainly cannot detect any flaw therein, *provided the general style of reasoning adopted by contagionists be admissible.* M. Forget, it appears, in his character of a "pupil of the Parisian school," denied the existence of contagion until his experience at Strasburg convinced him that "whether it be by inoculation of a specific virus (contagion), or by inspiration of an atmosphere vitiated in any other manner (infection)" he cannot determine, but "certain it is that the disease is *communicated* under certain circumstances sufficiently rare and as yet undetermined."

We now come to a much litigated point in the history of the typhoid fever of France, the relationship of the intestinal lesion to the disease itself. In his first edition, M. Louis contented himself with announcing the invariableness of the existence of this lesion, with inferring thence that it constituted the anatomical character of the disease, in other words the material proof of its existence, and with ascribing the intestinal symptoms to its presence. But others soon appeared less chary of inference, and at once elevated the lesion to the rank of a *cause.* These persons were not, however, precisely agreed among themselves as to the mode of sequence of the phenomena; hence two parties whose theories have been thus briefly and clearly stated by M. Piorry. The one maintains the series to be this: enteritis; ulceration; formation of sloughs and putrid matter; absorption of the latter; infection of the blood, and consequent general and secondary local phenomena. With the other, the correlation is as follows: absorption through the lungs of putrid miasmata; infection of the blood; follicular enteritis, attended with ulceration, and sometimes with gangrene; formation of putrid matter; absorption of these; general typhoid symptoms. M. Andral appears to have been unwilling to grapple with the question; nevertheless, so far as he ventures to go, he repudiates the idea of the causation of the disease residing in the intestinal change. "Admitting," he says, "that simple follicular enteritis is the starting point of many fevers, can everything be explained by it?"—No, concludes M. Andral, for "it has appeared to him that these fevers do not become severe, unless as a result of disturbance in the functions of innervation and hæmatosis." (Clin. Méd. ed. 3, t. i. p. 4.) The opinions of M. Cruveilhier are marked by vacillation and uncertainty. "How is it possible," he enquires, "to refer the general symptoms to a lesion of such limited extent?" . . . Is it not more probable that they depend upon a cause acting upon the entire system?" Yet further on we find him stating as "his conviction, after mature reflection, that the fever in follicular enteritis is symptomatic in the same manner as that attending eruptive diseases or pneumonia." (Anat. Pathologique, 7 Livraison.) M. Chomel is far from considering the intestinal change the essence of the malady; on the contrary, he infers that "all the symptoms, with the exception perhaps of diarrhœa, abdominal pain, and gurgling in the right iliac fossa," [a symptom to which, we may mention, M. Chomel attaches too high diagnostic value,] "are the expression of the influence of the disease on the whole system,—of the disturbance of the chief functions; and they are the effect of the disease itself rather than of the lesion of the follicles."

In our former article we stated, in the words of M. Louis, the inference drawn by him upon the point in question in his second edition, identically the same as that originally drawn. In the second, however, he considers the possible fallacy of arguing to the petty importance of the lesion from the want of proportion between the extent of this and the severity of the attending symptoms. Erysipelas of the face, he reminds us, is accompanied with very intense fever, at a period when the affected portion of skin is no larger than a shilling; the fever does not increase with the spread of the inflammation, nay more, it actually disappears occasionally, while the exanthema spreads to the trunk and extremities. This argument would be excellent, were it certain that the erysipelatous inflammation is the original cause of the fever; but as the latter precedes the former it cannot be its effect. It is further urged that the febrile reaction attending pneumonia is as intense when a very small portion as when the greater part of the lung is implicated. It must not be forgotten also that, besides the patches, the mesenteric glands and spleen are invariably affected in typhoid fever.—M. Forget's book is written with the express object of proving that the intestinal change is the efficient cause of the whole train of symptoms. He "cannot," he confesses, "comprehend the profoundness of the expression anatomical character," . . . as signifying a distinction from pathological character, and assumes the constancy of the intestinal lesion. Nevertheless he is constrained to admit that in some cases that lesion is secondary, inasmuch as it appears to result from a primary infection of the blood in epidemic typhus. The contradiction of previously-stated opinions involved by this admission he seeks to evade by the hypothesis that in typhoid fever of miasmatic origin, the "primary infection of the blood is utterly insignificant in comparison with the follicular enteritis," which is precisely the point in debate: in struggling to escape from a contradiction, he stumbles into a petitio principii. It has been said that the intestinal lesion cannot be the cause of typhoid fever, because typhoid fever (or better the typhoid state) may exist without the lesion, and the latter exist without the former. M. Forget observes that this argument is equally applicable to the opinion of those who regard the lesion as the effect of the fever, and illustrates this by a parallel case: "pneumonia may exist without fever, and fever without pneumonia; therefore the fever of pneumonia does not depend upon that pneumonia." This is fair enough, but M. Forget's whole argument leaves the matter just where he found it.

It is admitted even by the most determined upholders of the intestinal origin of the malady that disproportion is frequently observed between the symptoms and the lesion. Some of the counter-arguments on this point have appeared in our former article; it may be well to bring them together here. 1. In every disease the febrile reaction varies with the idiosyncrasy of the individual. 2. When the change in the intestinal glands is trifling, and the symptoms severe, the secondary lesions are to be taken into consideration. 3. Where the converse has been observed, the intestinal lesion has in many instances been found to be in progress of cicatrization. The general justness of this argumentation is not to be denied, nevertheless it does not remove the impression that the want of correspondence referred to is much more frequent in the instance of the typhoid affection of Paris than of the acknowledged phlegmasiæ.

Setting aside for a moment the question of causation, one scarcely less important arises; does the intestinal lesion once developed constitute the most important feature, and measure the danger, of the malady;—is it the point to which the therapeutical efforts of the physician should be directed? First, it is impossible to question its extreme importance in the former respects, were it only from the fact that one of its results, perforation, is the immediate cause of death in somewhat more than 9 per cent. of fatal cases.* But exclusively of this epiphenomenon, the French pathologists even of the eclectic school, Andral for example, recognize its importance. According to M. Cruveilhier, it "appears to regulate the course and phases of the malady." Secondly, in it those who advocate exclusively antiphlogistic treatment see the whole disease; but any argument founded on the success of such treatment loses its force from the fact that the mortality of the typhoid affection of Paris appears to vary to a very great extent under the same treatment, whether this be purely expectant, actively antiphlogistic, moderately antiphlogistic, purgative or symptomatic, and that, as has been already mentioned, the expectant appears to be the most successful mode of managing the disease.

From the materials of which the reader is now in possession, and from our own clinical observation, we believe ourselves entitled to draw the inferences which follow respecting the Parisian malady. Our *profession of faith* is deficient in the assured and dogmatical tone which would satisfy the enthusiasm of M. Forget, and we shall, we doubt not, be herded by him with those perverted mortals who, as he opines, "if TRUTH were one day to rise from the bottom of her well, would hasten to plunge her again to the bottom, in order to have the pleasure of looking for her still;" but for truth's sake we would bear the brunt of even bitterer sarcasm than this: 1. The various forms of continued fever occurring in Paris are all essentially varieties of the same malady, passing into each other in such manner that their separation is not admissible even in speculative nosology. 2. This malady, the typhoid affection or fever, is attended in the vast majority of cases with a change in Peyer's glands; error of diagnosis has probably swelled the list of cases of its absence. 3. It cannot be considered to be satisfactorily established as the primary cause of the disease, because, in addition to the fact of its occasional absence, the discrepancy between it and the symptoms is of considerably greater extent and more frequent occurrence than is the case with any affection in which the anatomical change is the acknowledged cause of the functional derangements. Less importance is to be attached to the want of direct proof that the lesion appears from the very first, because even in cases of pneumonia, for example, febrile disturbance frequently exists before the development of any local symptom; herein is manifestly involved the whole doctrine of the relationship of local maladies and febrile

* This estimate is founded on the following facts:

| | Deaths. | Perforation. |
|---|---|---|
| Louis | 55 | 8 |
| Bretonneau | 80 | 8 |
| Chomel | 42 | 2 |
| Montault | 49 | 5 |
| Forget | 44 | 2 |
| | 270 | 25, or 9·22 per 100. |

action. 4. The intestinal lesion probably exerts direct secondary influence in the production of the typhoid phenomena through absorption either of putrescent matter from the patches themselves, or of the morbid secretions in the bowel. But though probable this notion is far from being free from possible objection, because : *a*, typhoid phenomena arise without the existence of intestinal ulceration ; this, however, only proves that this is not the sole source of such phenomena : *b*, the formation of Peyerian ulcers in phthisis is not commonly attended with the evolution of typhoid symptoms ; however it is stated, or at least surmised, that in some instances it is so attended, (by Forget, a questionable witness the reader will possibly imagine,) and the different mechanism of the ulceration in both cases may be the cause of the difference in the ordinary result. 5. The lesion of Peyer's glands is primarily specific,* secondarily inflammatory. 6. The general muco-intestinal surface may be free from anatomical change. 7. The malady is attended with a peculiar proneness to membranous ulceration. 8. A first attack preserves the individual from a second. 9. It has rarely been observed after the fiftieth year; the reason of this is not clearly made out. 10. It is developed spontaneously and by contagion ; both under peculiar and imperfectly ascertained conditions : the latter we only admit with the proviso already stated.

The evidence proceeding from different parts of the continent is next to be considered. M. Louis himself has in his recently published work on yellow fever described some cases of typhoid affection marked by the same lesions of function and of structure as those characterizing the Parisian malady. The place in and the circumstances under which these cases were observed are important, for they show that the disease maintains its character at Gibraltar, and during the prevalence of an epidemic fever of still more destructive nature. The descriptions already referred to prove that the anatomical change follows identically the same course as at Paris, at Dresden (Carus), at Berlin (Böhm), at a village on the banks of the Danube (Buzorini), at Vienna (Rokitansky, Staberoh, &c.) From Böhm and Buzorini come the microscopical and chemical analysis of the exuded matter, whereby a precision is given to their statements, bearing in itself efficient testimony to their accuracy. But the German physicians recognize this lesion as the characteristic of one variety of continued fever only, that in which abdominal complication prevails, to which they accordingly give the title of typhus abdominalis; they do not regard it, when established, as the cause of the symptoms.

The epidemic fever which devastated Italy in 1816-17 was, as regards symptoms, to all appearance perfectly identical with that observed in this country. The symptoms are described with sufficient minuteness to justify this conclusion ; some of them indeed, for instance the eruption, with very remarkable precision. Hear Palloni's description : "Fra il terzo e il settimo giorno comparise alla cute una *eruzione* di macchie rossastre, puntiggiate, irregolari, e leggiermente scabre e rilevate (che costituiscono la vera eruzione tifoide), fra le quali talvolta si vedono sparse delle petecchie." (*Febbre Tifoide che ha regnato in Italia negli anni* 1816-17, p. 150.) Here among other points the distinction between

* It has been observed that all the irritating agents in the world will not produce follicular enteritis; though common enteritis may be produced artificially with ease.

the eruption and petechiæ, overlooked by an Edinburgh Professor of the present day, 's distinctly made. Unfortunately the cadaveric history of the epidemic is not detailed with similar accuracy; the abdominal cavity and its contents are stated to have been in some instances healthy; in others the "intestines and stomach were in some points more or less inflamed, empty, and contracted, or distended with mephitic gas, in other cases containing lumbrici." (*Storia della Malattia Febbr. Osserv. Nello Spedale Provisorio di S. Lucia, ann.* 1817, p. 119.)

The physicians of the United States have furnished some contributions on the subject of continued fever distinguished by considerable accuracy of research and narration. Among these observers Dr. Jackson holds a very prominent place. His experience is stated by himself in the following terms: "Since the work of M. Louis has been known to me, I have found that the continued fever, which is so well known to us, in this city at least (Boston), was the same as that which he has described. The symptoms are essentially the same, and the appearances discovered in the body after death are precisely the same. These appearances had been noticed here before, when the examination was so made as to disclose them. From 1833 our fever has been the same it formerly was, and *in every case*, where an examination has been made, the morbid changes have been found to be the same as described by M. Louis. In neighbouring places, a similar confirmation of the identity of the disease has been furnished from different sources. I may refer here particularly to cases which occurred in Lowell, and were reported by Dr. Bartlett, the learned professor of pathological anatomy in the Berkshire Medical Institute." (Report on Typhoid Fever, p. 8.—Boston, 1838.) The testimony of Dr. Jackson to the frequency of Peyerian lesion in America is the more valuable, as from another of his works we learn that "the discoveries made by Louis in respect to typhus were in contradiction to opinions which he had maintained and taught for many years."* Subsequent observations by Dr. Gerhard upon an epidemic continued fever at Philadelphia, observations to which we are about to refer more fully, led Dr. Jackson to believe that "there are, at least, two species of continued fever both in Europe and in this country; and further researches may very possibly show more." (Op. cit. p. 10.)

Dr. Gerhard returned to his own country prepared for the observation of its continued fever by a close scrutiny of that of Paris, and had already contributed a paper to the American journal, demonstrating the perfect identity of the two maladies in respect of symptoms and abdominal lesions (of the follicles, mesenteric glands, and spleen), when an epidemic continued fever came under his notice presenting characters to which he had been hitherto a stranger. His observations appear to have extended to 214 cases (120 men, 94 women), in "about 50" of which a post-mortem examination was instituted. The symptoms of this "typhus fever" appear to have accurately tallied with those usual in this country: the eruption wore the characters of that of Great Britain, there were neither diarrhœa,† abdominal pain, nor marked meteorism; and in one

* Memoir of James Jackson, jun. M.D., p. 78.

† At the close of the epidemic, when dysentery became prevalent in the district, diarrhœa occurred; but this was evidently a merely accidental epiphenomenon, and attended with softening, &c. of the mucous membrane of the *colon*.

case only was there lesion of Peyer's patches (and this of doubtful character) discovered, the mesenteric glands retained their healthy aspect, and the spleen was enlarged and softened in only half the cases. Dr. Gerhard believes this malady identical with the "typhus fever" of Great Britain:* we too believe it identical with our more ordinary form of continued fever. We are not acquainted with any series of post-mortem examinations instituted in the British Isles, wherein the proportion of follicular cases was so low as one in "about fifty." Dr. Gerhard's experience exhibits in this respect one extreme, while the other is supplied by the observations, to be presently more fully referred to, of Dr. Bright and Mr. Goodsir. Dr. Gerhard further believes the malady he describes, as well as the British "typhus fever," perfectly distinct from the continued fever of Paris on the grounds just pointed out, as well as the alleged fact of the extreme contagiousness of typhus, while "it is *very clearly proved* that the typhoid fever or dothinenteritis is not contagious." We have seen that M. Louis, after a careful investigation of the evidence respecting dothinenteritis, is persuaded that it *does* spread by contagion; and the unsteadiness of Dr. Gerhard's own creed appears from his saying elsewhere, "in some epidemics, we have strong reason to believe that dothinenteritis becomes contagious."

In approaching the branch of our subject which refers to the continued fever of this country, we feel considerable hesitation, because we shall have to examine with unusual severity the productions of estimable men, and because we feel diffident of our own powers to influence general opinion materially upon a question which admits of very strong argumentative support whichever side be espoused. Let not the reader charge us however with the pretension to *decide;* we offer the result of our enquiries, impartial as we believe them to have been, as a simple contribution to the settlement of the question.

We can scarcely be accused of defective patriotism, especially as the same is true of other countries, if we set out by stating that the accounts published respecting the typhoid fever of these islands were, until a very recent period at least, utterly devoid of scientific precision. It is manifest that their predominating characters were vagueness of description, boldness of assertion, and deficiency of anatomical details: their epigraph might have been, *Viel Irrthum und ein Fünkchen Wahrheit.* And this spark of truth disserved rather than promoted the cause of progress,

* "The typhus fever," observes this writer, "which is so common throughout the British dominions, especially in Ireland, is not attended with ulceration or other lesion of the glands of Peyer; I mean that this lesion when it occurs, is merely accidental or a *complication* not occurring in the ordinary course of the disease." Yet it is in the very same page affirmed, that "an *error* frequently committed by the British physicians is, that they regard this affection (dothinenteritis) as a mere *complication* of their ordinary typhus, or a modified form of it." (p. 292). So that what is error with us, becomes truth when announced by Dr. W. W. Gerhard. There is a want of precision in some, indeed we might say in all, the numerical details of this writer, at which we felt no little surprise. What accurate notion is, we would ask, derivable from such statements as these: "the mortality among the cases that were treated by us from the beginning was *not great;* but the total loss of patients admitted at an advanced period of the disease, many of whom were moribund, was very considerable, *about* one in three."

by giving collateral authority to the multitude of errors with which it was associated. From such portion of our literature, as is thus characterized, we can expect no available evidence on the topic under consideration, and are obliged to come to a very recent period in search—even here the search is commonly fruitless—of documents bearing upon them the stamp of close observation and correct description. The works published of late years may be classed under four heads; of essays in Cyclopædias, ex professo volumes, papers containing more or less accurate reports of cases, and essays recording the numerical analysis of symptoms and lesions in a certain number of cases. We cannot of course examine all of the very numerous productions referrible to these categories which have appeared; we must content ourselves with one or two specimens of each.

Dr. Christison's essay is the production of a determined essentialist and of one denying that the Peyerian lesion can be considered otherwise than as of unusual occurrence in the town where he practises. The latter is at least an inference to which the general tone of the essay leads, though, as is shown by the following passages, its author has not announced the opinion in such terms as to obviate the chance of its being deemed one espoused upon insufficient grounds. At page 139 we read that the intestinal lesion "is found in the Edinburgh Infirmary only often enough to make the pathologist acquainted with its phenomena and *keep him in mind of its existence;*" at page 121 it is said that "the appearances of local disorder are now well enough known to every scientific physician and are *frequently seen* by practical men;" while at page 153 we learn that "in the fever of Edinburgh serious bowel affections are in general *very uncommon.*" But the tendency to contradiction apparent here is trivial in comparison to that exhibited in another part of the essay: Dr. Christison devotes a section to the "*anatomical characters*" of typhoid fever, while his entire previous matter is designed to show that the malady has *no* anatomical character, that there is no constant attendant organic change. And whence derives he his anatomical descriptions; from his own experience of the disease as it exists at the Edinburgh Infirmary?—No, but from the volumes of M. Louis. In other words he describes a given disease by borrowing from the description of another, which other he maintains to be perfectly different therefrom. Dr. Christison cannot shield himself from the charge of self-contradiction by maintaining that the changes of which he borrows the description are secondary lesions of typhus; even to this defence he can hardly have recourse, for he tells us (p. 153) that "most of them [the anatomical changes described,] it is reasonable to conclude, are secondary not to fever in general, but to one of its secondary disorders—inflammation of the mucous glands of the intestines," which "inflammation," be it remembered, is declared to be a "very uncommon" attendant on the Edinburgh continued fever. For further illustration of this author's doctrine we shall here transcribe, with the comments they suggest, certain other passages of his essay:

"The views entertained of fever in the abstract have not been rendered clearer since the time of Cullen." (p. 114.)

We are not to understand from this that Dr. Christison teaches us to believe in, inter alia hujusmodi, the spasm of the extreme vessels; but to the division of continued fever into three species he reverentially adheres."*

"M. Louis has been led to the conclusion that the typhoid form at least of fever is always owing to inflammation of the glands of the intestinal mucous membrane." (p. 120.)

Here is a treble inadvertence. The first line of M. Louis' *avertissement* states, what the whole work proves, that the term typhoid fever or affection embraces all the species of continued fever of nosologists. Secondly, far from asserting that typhoid fever is *always* owing to inflammation of the intestinal glands, he does not in any instance maintain such causation. Thirdly, in applying the unqualified term inflammation to the state described by Louis, his opinions are misrepresented; the followers of Broussais are at this moment quarrelling with him for proving the malady to be of *specific* nature.

"It may in particular be doubted whether the disorder described by Bretonneau and Louis is not a local disorder which simulates fever." (Ib.)

The meaning of a local physical change simulating a group of general functional disturbances is not very clear; but inasmuch as this "local disorder" is said to correspond to "fever" in France, it follows that there is no such thing known in that country as "essential fever." Rather a curious inference to flow from the writings of a professed "essentialist."

"Physicians at Paris have plainly formed their conclusions regarding the nature of fever in the abstract from observing the characteristics of a single form *rarely presented in the epidemic shape.*" (p. 122.)

The description of Roederer and Wagler was drawn up from an epidemic at Göttingen; Cruveilhier observed the disease epidemic at Paris in 1814-15, and at Limoges in 1816-17; Gendrin's descriptions are altogether founded on epidemics of the disease; so are those of Putegnat; Pellerin described the intestinal lesions from an epidemic which reigned at the Salpêtrière in 1814, at a time when those lesions had not been generally recognized; Ruef described an epidemic of it at Bischofsheim in 1832; Mistler at Andlau in 1833; Lombard has frequently known it epidemic at Geneva; it occasionally becomes epidemic at Paris (as in 1835), &c. &c. Be it observed that, except *probably* in some cases occurring at Paris in 1814, the iliac lesions invariably existed; nor has there been a single epidemic in the provinces of late years wherein these were not discovered.

At p. 122, note, the author states his impression that "M. Louis' opinions have undergone some change since the publication of his last writings on the subject of fever," because he admitted lately to Dr. Christison that a continued fever, differing from the French typhoid, prevails in England."

We really can scarcely understand the existence of *change;* for in the "writings" referred to, M. Louis made no shadow of allusion to the typhoid fever of any country but his own.

* Well does Dr. Jackson observe (Opusc. cit. p. 10): "First, such distinctions between continued fevers, as those of synochus, synocha, and typhus were not shown to exist in nature and were in truth grounded on men's fancies; and, secondly, those names were originally significant, not indeed of different qualities in nature, but of men's notions in regard to the different natures of diseases."

Among the chief distinctions between idiopathic local inflammations and inflammation attending primary inflammatory fever, is the alleged circumstance that in the latter "the inflammation arises consecutively to the fever." (p. 129.)

This is precisely the point which Dr. Christison was called upon to prove, for up to the present day we know not that such demonstration has ever been given. If Dr. Christison had said "*appears* to arise," he would have spoken in conformity with observation. But had he done this we should have told him that the same is true of every one of the acknowledged phlegmasiæ,—that there is not one of these the development of which, as cognizable by ascertained local symptoms and physical signs, may not be preceded by a febrile movement of more or less duration. Whether an anatomical change have in reality developed itself, though to so limited an extent as to produce no appreciable symptoms, is a problem in the present state of knowledge insoluble.

"Hence of 690 cases crisis took place in 470 on critical days, in 52 on the subsidiary critical days, and in *only* 108 on the days which are considered non-critical." (p. 133.)

Let us enquire whether these figures supply satisfactory reasons for assenting with Dr. Christison to the notion of "critical days." If we found that these alleged critical days were two or three in number out of twenty, the evidence would be imposing, nay conclusive; but what is the fact? Why that these critical days are no less than ten out of twenty; and that hence the chances of favorable termination are equal on the so-called critical and non-critical days, independently of any mysterious influence exercised by septenaries and multiples and submultiples of the same. Again; we find 34 cases noted as terminating on the sixth day; 129 on the seventh; and 26 on the eighth: now let the reader consider the difficulty of fixing within twelve hours the time of origin of such a malady as fever, (which not unfrequently runs a latent course for a variable period;) next let him fancy the invincible tendency in any mind, duly impressed with the mystic importance of the seventh day, to consider patients a little worse on the sixth or a little better on the seventh than common observation might adjudge, and then let him answer us whether it is not probable that day the seventh has been given a Benjamin's portion of the recoveries, to the detriment of the sixth and eighth especially, but possibly of every one of the subsequent days.

"The third variety of petechiæ presents more or less numerous spots," &c. &c. (p. 141.) Here follows a description of the rose-coloured spots of typhus.

These spots we need hardly remind the reader are not petechiæ at all: to confound a cutaneous efflorescence with a cutaneous and subcutaneous hemorrhage exhibits rather lax notions of anatomy.

"By many French pathologists one variety, the diffuse pale *petechia* (typhoid eruption), is thought peculiar to dothinenteritis." (p. 142.)

We know not who these pathologists may be: Andral, Cruveilhier, Chomel, Barth, Piorry, Bouillaud, Rostan, &c. admit that similar spots occur in other diseases, though much more rarely. Chomel even goes so far as to affirm that the number of papulæ must be considerable to give them any value in the diagnosis of typoid fever.

Having described miliaria as consisting of "*spots........filled* with a clear fluid," Dr. Christison adds, "sometimes they seem connected with sweating, break out immediately after a diaphoretic *crisis*, and then constitute what authors term *sudamina*." (p. 143.)

This is not perfectly correct; Louis and Chomel, who have the most carefully described the epidermal vesicles under the title sudamina, are precisely those who deny their connexion with perspiration; whether they do this correctly or not is another matter.

Before taking leave of Dr. Christison's essay on fever, we cannot avoid expressing our very sincere hope that he will regard the close criticism to which we have ventured to subject some of his opinions and statements as evidence of the importance we attach to any production of his. His acknowledged talents and eminent position render it most important for the cause we advocate that his doctrine should be proved to be not unopen to objection.

Dr. Hodgkin commences his chapter on fever by observing, that "the frequent occurrence of inflammation and ulceration in the inferior portion of the ileum in cases of fever is confirmed by the testimony of all the best and most accurate pathologists, who of late years have investigated the morbid appearances connected with the febrile state . . . . . It is the glandular apparatus, and more particularly the aggregate glands which are the principal seat of inflammation." (p. 459.) In the following page Dr. Hodgkin speaks of Louis as connecting their inflammation "with fever, or at least a *particular form* of fever, to which he gives the name of affection typhoïde." The error implied by this sentence has been already pointed out.—At page 463 we find the phrase "Louis, who considers *typhoid symptoms* as pathognomonic of inflammation of the aggregate glands." It is not easy to see how Dr. Hodgkin could have conceived M. Louis to maintain anything so utterly at variance with all experience: Louis is perfectly aware, as his volumes show, that typhoid symptoms (l'état typhoïde) may supervene upon almost every affection to which the system is liable, that pneumonia, that inflammatory affections of the urinary passages, of the renal textures, of the veins after external injuries, amputation or otherwise, &c. may be thus attended.—"If we admit the facts which I believe Professor Louis to have drawn up with conscientious accuracy, I do not see how it is possible to evade the conclusions at which he has arrived. . . . . . It appears to me to be altogether presumable that the inculcation of the doctrine, that in fever the glands of Peyer at the termination of the ileum are always in a state of [special] inflammation; and that, on the other hand, these glands are never affected but in cases of fever, may lead many to adopt the conclusion, that the inflammation of the aggregate glands in question really constitutes the essence of fever," (p. 464.) Are we to infer that Dr. Hodgkin would suppress what he believes to be a truth, lest mistaken persons should draw false or questionable inferences from it? But both passages are important: the first for an obvious reason; the second as showing that Dr. Hodgkin at least acquits M. Louis of affirming the intestinal change to be the cause of the malady.—"The name *gastro-enteritis*, which has been introduced as synonymous with, but more correct than, fever, and which Louis himself has adopted, at least in the title of his work, to convey the same idea." (p. 465.) There is here misapprehension: on the title-page of M. Louis' book (1st edition) we find the words, "Researches upon the disease *known under the names* of gastro-enteritis, putrid, adynamic, ataxic, typhoid fever," &c. &c.; the author meant simply to show by this title, that his researches referred to a malady well known to his countrymen by the recently devised title of gastro-enteritis, and nothing more. Had the phrase stood, "Researches upon gastro-enteritis," the matter would have been very different; the truth is, that M. Louis never uses the term except when engaged in particularly demonstrating its total inapplicability to the affection he describes.—"Does it not seem reasonable and consistent with analogy," enquires our author somewhat further on, "to presume that . . . on the one hand there may be symptoms of general disturbance of the system prior to, and often disproportioned to, the inflammation of the elliptical patches; and, on the other hand, that these patches may be deranged, with comparatively little general disturbance?" (p. 466.) Why, no one questions this; the very cases of M. Louis prove it, and he himself believes (which destroys the force of Dr. Hodgkin's objection still more completely) that even in the instance of the phlegmasiæ "there is general disturbance prior to the inflammation." Can anything be more strange than the habit indulged in by some writers of attempting to establish doctrines upon analogical and argumentative grounds, when their soundness on the contrary might be immediately and satisfactorily settled by an appeal to direct observation?—At p. 477 we find Dr. Hodgkin referring to the "striking accordance between the results of Louis and those of other investigators, who, with ample opportunities, have given minute attention to the subject;" among others Dr. Bright. In Dr. Hodgkin's own experience, "the derangement of the aggregate glands has certainly been detected in most of the fatal cases of fever which he has examined," (p. 481.) "He is persuaded," however, that in some cases "presenting the characteristic symptoms of fever during life, he has found the aggregate glands, so far from being severely deranged, barely discernible, whilst the cerebral or thoracic derangements have been very considerable." (Ib.) Dr. Hodgkin has seen ulceration of Peyer's glands in cholera, and "in the body of a little girl who had died, not from scarlet fever, nor of the *ordinary symptoms* of continued fever, but after a much more lingering malady [what malady?] terminating in an acute attack, in which profuse hemorrhage from the bowels was the most remarkable symptom." (p. 520.) Where is the *proof* that this was not actually a case of continued fever? This author indulges in much speculation respecting the alleged verisimilitude of the symptoms attending "Cynanche maligna," and "inflammation of the patches of Peyer;" the only importance of this speculation arises from its involving the notion that the Peyerian lesion, when it exists in typhoid cases, is the cause of the fever, a notion which a few pages back appeared so noxious to Dr. Hodgkin, that he would have suppressed a truth in order to exclude the chance of its being entertained. By the foregoing quotations, the ideas derived by Dr. Hodgkin from experience in respect of the relation of continued fever and Peyerian lesion are made apparent. He would perhaps desire us to add, that he "imagines fever to depend on the suspension, or at least very considerable interruption of that process by which, during health, the various parts of the system are continually undergoing a change." (p. 490.) Others might imagine this suspension or interruption the effect, instead of being the cause of fever.

A systematic work by Dr. Roupell (A short Treatise on Typhus, Lond. 1839,) deserves reference in this place (we noticed the volume on its publication, vol. VIII. p. 428,) from three circumstances only: the frequency with which the author observed the papular typhoid eruption,* the fact that his anatomical details are taken from Louis and Chomel, and his reference of the typhoid symptoms to intestinal phlebitis.

Dr. Bright, incomparably the most accurate observer of the anatomical changes of the continued fever of London, writes thus: "Whatever may be the primary condition of the febrile attack, there can be no doubt that early in the disease, *not only in the season of which I have spoken* (autumn of 1826), *but almost always,* the intestinal canal is irritated, and that this irritation keeps up all the bad symptoms, becomes the *chief object* to which the practitioner should turn his attention, and is not unfrequently at last the immediate cause of death." (Reports, vol. i. p. 178, London.) To the early manifestation of intestinal disease he bears witness in the following passage: "In some cases the symptoms of irritation in the bowels have followed very quickly after the first indications of fever, almost always before patients have been admitted into the hospital, which is seldom within the first week, and frequently not within the first fortnight after the attack." (p. 179.) Ten cases are given, in every one of which the special lesion of the patches, as also of the mesenteric glands, when these are mentioned, displayed itself under the precise aspects observed at Paris; the phenomena of distension with yellow cheesy matter, sloughing, ulceration, perforation and cicatrization, were all undistinguishable in character from the corresponding changes described by Louis.

The most valuable, indeed almost the only English work belonging to the third class is the essay of Drs. Henderson and Reid, in the Edinburgh Journal. The description of symptoms by the former gentleman is founded on nearly 200 cases which came under his particular care between October 1838 and June 1839, a period at which the malady prevailed epidemically. Dr. Henderson "has had recourse, as far as the records enable him, to a numerical analysis of the most important symptoms." Among the introductory remarks, we find it observed that "the difficulty of forming a satisfactory diagnosis in many instances, in which the disease terminated within six or eight days," was distinctly felt;—an excellent comment on Dr. Christison's readiness in accepting the "critical day" cases. "Convalescence was gradual, sudden improvement and speedy recovery, in connexion with abundant secretion from the skin, bowels, or kidneys being extremely rare;" so much for so-called "crises." We have changed in the following brief abstract, the order in which the symptoms are described by Dr. Henderson, for the purpose of facilitating comparison with M. Louis' results, as already given; the comparison can-

* Dr. Roupell appears to believe that in terming typhus an exanthematous disease he has effected a novelty: "Il tifo," says Palloni, "così detto petechiale, quale oggi regna fra noi, *è una febbre esantematica.*" (Op. cit. p. 149.)

not, however, be as close as desirable, more especially because Dr. Henderson has commonly failed to note the number of cases in which each symptom was investigated.

Distinctly spontaneous diarrhœa appears to have occurred in 8 only of 149 cases, at a variable period of the disease, not exceeding six or eight stools per diem. Among 104 females 31 had costive bowels at the time of admission, 73 had them in an easy state, 28 of whom had taken purgative medicine; of 45 men, the bowels were costive in 19, open from medicine in 15, and moderately so without medicine in 11. Forty-six suffered from abdominal pain, 21 in the epigastrium, 19 over the whole extent, 2 in the left iliac region, none in the right, 3 in the left hypochondrium, 1 to the right of the umbilicus. Eight patients had slight meteorism, attended with general abdominal tenderness, and in one case with diarrhœa. Twelve had nausea and vomiting, chiefly at the beginning; in 5 they were accompanied with abdominal pain and tenderness. 104 of 108 females had cephalalgia, 92 of them at the outset; 49 of 51 males had that symptom; its mean duration in 29 cases was ten days; in a few cases it prevailed during the entire course of the complaint. Twenty-five females, or less than one fifth, had delirium, the mean period of origin being the eleventh day, in 3 cases, before the ninth; 23 of 66 males, or more than one third, suffered from it also, the tenth day being the average day of its appearance. Thirteen females and 3 males only are noted as having had somnolence; it appeared later than delirium. Fifteen females and one male had deafness; spasmodic symptoms of various kinds were noticed. An exact account was kept of the state of the tongue in 100 female cases. "It very early became covered with an increased and altered secretion, . . . . in 73 cases the tongue was dry at one period of the disease, in 27 it continued moist throughout, and in several of these was at the same time almost quite clean, in cases too which were not always mild, for one with the tongue in this clean and moist state died on the 13th day."

From an extremely minute and elaborate description of the typhoid eruption we extract the following particulars: of 130 cases of both sexes in which this was sought, 108 presented it; in 22 it was not found, but 6 of these were not admitted till between twelve days and three weeks from the outset, and may have had it before admission. The greater number of the 16 non-eruptive cases were slight, 1 only severe. In several of the other subjects petechiæ were detected. Four forms of the eruption, which it is unnecessary here to refer to particularly, are described. The *colour* appears to have varied with that of the skin, from the dusky red of dark persons to the light pink of the very fair. The upper part of the chest, the submammary regions and sides of the abdomen were the most favorable situations for observing it, but it occurred also on the extremities. Its first appearance was witnessed in 12 cases, from the third to the thirteenth days. Its development after the eighth day must be extremely rare, for though many patients were admitted after the first week the formation of the efflorescence was only observed three times after that day. Its development usually took place by successive daily additions; from two to four days appears to be its ordinary duration in the complete state, but a duration of five or six was in some cases observed; four or five were occupied in the fading, the whole duration commonly equalling nine or ten days. Its abundance is of unfavorable augury—a fact ascertained by comparing the number of deaths occurring in cases where it had been abundant, or the contrary, as well as the mean duration in cases of recovery; thus:

| | Eruption copious. | Scanty. | | Copious. | Scanty. |
|---|---|---|---|---|---|
| Cases . . . . | 65 | 25 | | 55 | 18 |
| Deaths . . . | 13 | 3 | Duration ... | 13⅔ days. | 11½ days. |

Among appearances produced by ecchymosis, purple *petechiæ* were frequently present in the second week; a single *vibex* in one case only. *Sudamina* were observed in three instances only, on the tenth, eleventh and thirteenth days. Twenty-three persons had thoracic symptoms; of 25 men, 5 had cough or slight pain in the chest, 18 bronchitis, with cough, expectoration, and the usual catarrhal ronchi; of 48 women, 15 had slight cough or pain on deep inspiration, 33 bronchitis. No example of pneumonia occurred. The *ages* of 161 subjects were as follows:

| | Males. | Females. | | Males. | Females. |
|---|---|---|---|---|---|
| 60 | — | 1 | 20 and below 25 | 1 | 13 |
| 50 and below 60 | 3 | 3 | 10 ,, 20 | 15 | 41 |
| 40 ,, 50 | 5 | 10 | 5 ,, 10 | 5 | 10 |
| 35 ,, 40 | 1 | 7 | 4 ,, — | — | 1 |
| 25 ,, 35 | 12 | 33 | | | |

As an approximation to the mortality, Dr. Henderson gives 1 in 11½, for females, 1 in 7⅓ for males, or 1 in 9½ for both sexes: at least one fourth of the subjects denied exposure to contagion, and ascribed their illness to cold, while about as many could not refer it either to contagion or cold, so that in half the cases no plea for assigning contagion as the cause could be obtained. Nevertheless, "whether any examples of fever actually occurred without there having been previous intercourse, Dr. Henderson had no means of ascertaining,"—a phrase announcing the determined contagionist.

A description by Dr. Reid of the morbid changes discovered in 47 subjects cut off by fever, follows Dr. Henderson's contribution. The value of these details is diminished by their not referring to individuals observed by Dr. Reid during life; but in respect of our present enquiry this is a point of very secondary importance. Dr. Reid refers to the symptoms noted in the register by the physicians or the pupils.

Of 43 subjects whose *encephalon* was examined, 25 presented an abnormal quantity of serum within the cranium; the vessels of the brain were in all the cases except one well filled with blood, and in many sections of its substance appeared studded with red points; in one only was there a slight degree of softening; it was universal in this instance and occurred in hot weather. Dr. Reid enters into precise details respecting the character of the cerebral effusion and its relation to symptoms; and it appears from them that no symptoms can be distinctly ascribed to such effusion. The *lungs* were examined in 43 cases with the following results:

| | | |
|---|---|---|
| Scarcely engorged even at the depending parts | | . 9 |
| Engorged at depending parts | | . 4 |
| ,, | with frothy œdema | . 6 |
| ,, | with tubercles | . 2 |
| ,, | with old pneumonia | . 1 |
| ,, | with mucous effusion in the bronchi | 9 |
| Congested at posterior and middle parts | | . 10 |
| Recent pneumonia | | . 1 |

Dr. Reid was extremely careful in admitting pneumonia among the lesions of typhus, being well acquainted with the kind of error of which that inflammation occasionally becomes the subject. "When fever is prevalent," he observes, "cases of latent pneumonia in an advanced stage and attended by typhoid symptoms are very readily mistaken for continued fever, unless the attention of the physician be particularly directed to the chest. I examined the bodies of three patients within a short time, who had been sent into the infirmary as cases of continued fever, but, from the appearances observed on dissection, there could be little doubt that they were cases of latent pneumonia which had gone on to the third stage, with very little of the usual pectoral symptoms."

The abdominal organs were examined in 41 cases. The state of Peyer's glands in these cases is exhibited below.

| | | |
|---|---|---|
| Apparent and distinctly defined in 24, of which . . | Grayish and dotted with dark spots 22 | Not so 2 |
| | Distinctly elevated 4 | Not so 18 |
| | Very distinctly elevated with traces of ulceration 2 | No traces of ulceration 20 |
| Indistinctly defined and scarcely visible . . . . | | 6 |
| Not distinctly to be recognized with the naked eye . . . | | 11 |

The solitary glands at the lower part of the ileum were distinctly visible in four cases only. The mesenteric glands were enlarged in two cases, in one softened; some of these enlarged glands were one inch and a sixth in length, the same in breadth, and half an inch thick. The grayish discoloration with black dots (likened to a *barbe noire récemment rasée* by some French writers) has been met with by Dr. Reid in various other diseases though not with equal frequency. In the cases in which the patches were visible the average duration of the disease was nearly fourteen days, in those in which they were invisible nearly twelve.

In 101 cases of continued fever, examined by Dr. Home, the patches of Peyer are described as being well defined or enlarged in 29; in 7 of these 29 there was ulceration; in 2 of these 7 perforation. It is extremely remarkable that Mr. Goodsir, residing at Anstruther, only thirty miles from Edinburgh, who has in the course of five years examined the bodies of 10 persons dying of fever in his neighbourhood, found the elliptical patches of Peyer and the solitary glands thickened and ulcerated in every instance, and one of the latter perforated in four cases. The greater number of fatal cases occurred in individuals of from 13 to 20 years of age. The series of changes in the patches is very closely described by Mr. Goodsir.

The only other abnormal appearances in the intestines of the 41 subjects were, in 1 a few spots of ecchymosis in the jejunum and upper part ofthe ileum; in 1 a morbid gray elevation adhering to the mucous membrane; in 1 some old cicatrices at the lower part of the ileum with circumscribed red spots; in 1 small depressions, with surrounding redness, of the cæcum and colon; in 1 similar depressions without redness in the cæcum and ascending colon; in 1, numerous small red patches of transverse and ascending colon, with slight sponginess and elevation of the parts reddened. Of 6 patients dying from other diseases, during convalescence from fever, 4 were examined: they had died on the 31st, 27th, 20th, and 33d days.

In the 3 first there were no cicatrices at the lower part of the ileum, in the other the patches were uniformly dark red through the entire tract of the ileum, distinctly defined and elevated, irregular on the surface, and at one or two points presented traces of incipient ulceration. In 9 cases only of the 24, in which the patches were distinctly visible, were there any abdominal symptoms during life, and in 3 of those 9 the symptoms could not be referred to the Peyerian lesion. The condition of the stomach in the 10 cases out of 41 in which alone that viscus presented any deviation from the normal state was as follows;

| | |
|---|---|
| Mucous membrane thickened and mammillated at pyloric and middle parts | 3 |
| Slightly mammillated near pylorus, softened at splenic end . . . | 1 |
| Splenic end corroded and largely perforated . . . | 1 |
| Universally thickened, indurated, and mammillated . . . | 1 |
| Presenting numerous rounded superficial depressions, with defined margins, without thickening or redness . . . . | 1 |
| Red and friable, and covered with a layer of mucus towards the pylorus; presenting also several small depressions with bright red margins . | 1 |
| Splenic end acted on considerably by gastric juice . . . | 2 |

The *spleen* was of all the organs in the body that most frequently found in an abnormal state, being generally larger than usual, soft, and in some cases almost diffluent. Six subjects had Bright's disease of the *kidneys*; the *liver* was the seat of old disease in five, as was the *heart* in three of forty-three cases. In one case there was recent inflammation of the *larynx*. In all the cases the blood appeared to be fluid, or nearly so, in the large veins; but in several, small soft coagula were found in the right cavities of the heart.

From Dr. Anderson's numerical analysis of cases of continued fever, observed by him at the Glasgow Royal Infirmary, we extract the following table of the lesions of the gastro-intestinal mucous membrane in seventy-four subjects:

| | Soft. | Follicular. | Peyer's Glands. | Solitary Glands. | Brunner's Glands. | Ulcers. |
|---|---|---|---|---|---|---|
| Stomach . . | 57 | 21 | — | — | — | — |
| Duodenum . | 18 | — | 1 | 2 | 4 | — |
| Jejunum . | 38 | — | 62 | 4 | — | — |
| Ileum . . | 45 | — | 68 | 26 | — | — |
| Cæcum . . | 45 | — | — | 11 | — | 5 |
| Colon . . | 38 | — | — | 27 | — | 4 |
| Rectum . . | 21 | — | — | 10 | — | 1 |

Some foreign physicians visiting this country have formed and recorded opinions respecting the character of its typhoid or continued fever. M. Lombard, struck with the almost identity of the symptoms observed at Dublin and at Geneva, while follicular lesion was absent in two subjects examined by him in the former town, in none dying in the latter, was induced to infer (in a first paper wherein he cannot allow our typhus and his typhoid fever to be "specifically distinct") "that typhus fever is more a general disease affecting the whole constitution than a malady depending on any local inflammation or any local change of structure," and that various circumstances "serve to impress upon this general disease a tendency to associate itself with and produce various local ailments." (Dub.

Journ. of Med. Science, vol. x., p. 23.) In a second essay at settling the pathology of fever, " a change comes o'er the spirit of his dream,"—typhus is perfectly distinct from typhoid fever, it is an Irish disease, being disseminated through Great Britain by Irish emigrant labourers; and further, it is identical with the gaol and camp typhus of French writers. This total change of opinion in the short space of less than a little month, is particularly instructive. The last version involves the idea of dissimilarity between typhoid fever and camp typhus, a notion repudiated, as we have seen, by Louis. That the Irish peasantry can be justly charged with being the originators and propagators of the continued fever of Great Britain is utterly disproved by the statistical enquiries of Dr. Cowan* and the logical inferences of Dr. Staberoh.† " Expliquez moi, mon cher," said a French to an English diplomatist, " expliquez moi, je vous prie, votre système financier; mais dépêchez vous—*je n'ai que cinq minutes.*" M. Lombard's five minutes were excellently well spent.

Dr. Julius Staberoh (Dub. Med. Journ. vol. xiii. p. 426,) observed a considerable number of patients in Scotland and Ireland, and discovered in a certain number of instances, (the proportion varied at different times,) intestinal disease with the precise characters, from exudation into the sub-mucous membrane of the patches to perforation of the gut, attending the typhoid fever of Paris. Dr. Staberoh insists most strongly upon the secondary and asthenic character of the inflammation of the mucous tunic of the patches, and its dependence upon the sub-mucous exudation. He regards the disease when attended with Peyerian lesion as specifically different from " typhus" when not so attended: " scarcely any of the medical men of Liverpool and Manchester," he observes, " were disposed to adopt that specific difference between typhus fever and gastro-enteritis or dothinenteritis," [just as if the two latter stood in the same relation to typhoid fever,] " which, *I am very sorry to say*, will perhaps still a long time prevent the medical men who have not the opportunity of judging both forms, when they prevail epidemically and endemically, from taking a general view of continued fever." It would be impolite and ungrateful not to acknowledge the kind commiseration evidently felt by Dr. Julius Staberoh for the benighted condition of such among our countrymen as are blind to the " specific difference," which he himself is fortunate enough to see so clearly: we wish him in return, as that which it appears to us would benefit him most on the present occasion, the least possible share of that mental amaurosis he pities us for possessing.

M. Louis, in the section of his work to which we promised to return, expresses his opinion that " observation has proved, that if examples be

* Vital Statistics of Glasgow.

† The author of a " Twelvemonth's Campaign with the British Legion," in describing the fatal effects of the epidemic typhus, which attacked that body in January, 1836, furnishes abundant evidence against the notion that the constitution of the Irish is adapted in any peculiar degree for the reception and propagation of typhus. He states that " the English and Scotch suffered extremely, while the Irish Brigade, composed of the 7th, 9th, and 10th regiments, enjoyed a perfect immunity;" and adds, " had the whole been composed of Irish, instead of losing nearly 1000 men at Vittoria, we might not have lost 100. In spite of all their hardships, the severity of the weather, the badness of rations, and total want of pay, the Irish lived, throve, and grew fat, as if in the midst of clover." (See Geary's Report on Typhus, Dub. Med. Journ., July, 1837.)

daily seen in England of an acute disease apparently similar to the typhoid affection, wherein nevertheless the patches of Peyer are healthy, the reason is, that two diseases exist in England, the symptomatology of which, though presenting many points of resemblance, is far from identical; that one of these is constantly attended with the special lesion of the glands of the ileum, whereas that lesion never exists in the other." The cases upon which this opinion is more particularly founded are thirteen in number, and were collected in the metropolis by Dr. Shattuck. Seven of these cases relate to patients, of whom the two oldest were 41 years of age, the others from 20 to 24. These patients had precisely the symptoms of the typhoid affection of Paris, diarrhœa, abdominal pain, meteorism, and a rare pale pink lenticular eruption; one of them died and presented the characteristic lesions of the glandular patches, of the mesenteric glands, and of the spleen. The other group of subjects were aged from 28 to 58, had a certain degree of stupor, febrile reaction, a pulse without marked development, occasionally delirium, and considerable weakness, but they had neither abdominal pain nor diarrhœa, unless after the exhibition of purgatives, rarely meteorism, which was trifling when it existed, and instead of the Parisian form of eruption, one characterized by its abundance, its universality, its incomplete disappearance under pressure, its reddish-brown colour, non-prominence, and persistence after death, absolute effusion of blood having occurred into the cutis and subcutaneous cellular membrane. Four of these subjects died: the glands of Peyer, as well as those of the mesentery, were perfectly healthy; the spleen enlarged and softened in one case only. Symptoms justifying localization of the malady were here evidently wanting, and no abdominal lesion existed: the mean age, also, of the two classes of subjects was different. The inference deduced from these cases by M. Louis is, he considers, confirmed by the relation of Dr. Gerhard already referred to; and from the whole he further concludes: "Not only has *typhus fever* not its seat in the digestive organs, as the typhoid affection, but it is impossible to assign it any seat; in the existing state of knowledge it has no anatomical character; and we are thus led naturally to affirm with M. Valleix, that *typhus fever* might be considered as an essential fever."

The essay of Dr. Stewart was undertaken with the view of "developing *some* of the leading features of difference," which in his estimation prove that "the two varieties of fever" (the continued fever of Paris and of this country) are "totally different diseases." He successively considers, "1, the probable origin of the two affections; 2, their proximate causes; 3, their course; 4, *some* of their symptoms; 5, *some* of their anatomical characters; 6, their treatment." Premising that the word some, for the italics of which we are answerable, means every symptom and lesion which Dr. Stewart has been able to discover corroborative of his views, we proceed to accompany him in his parallel.

I. Dr. Stewart points out the mass of evidence ordinarily adduced to show, that the accumulation of typhus patients within a small space causes the production of the disease in those brought within the sphere of such accumulation, and observes that circumstances have occurred wherein the *generation* of typhus can only be ascribed to the action of the animal effluvia thrown off from the bodies of individuals unaffected with fever, but cooped up together within a narrow space, and otherwise diseased, &c. Dr. Stewart next denies that a similar mode of origin of the continued fever of Paris can be supposed to prevail. In respect of concentrated *febrile* effluvium spreading the disease, we beg to enquire of Dr. Stewart where he has found the facts warranting his assertion; the French possess neither "fever hospitals" nor "fever wards," and without the aid of these, how can the necessary comparison be established? In regard to concentrated *non-febrile* human effluvium *generating* typhus and not generating dothinenteritis, Dr. Stewart's inference is, to say the very least, unwarranted by the facts before the world. We might tell him that the very cases of typhus alluded to by him as thus putatively generated (and we are by no means disposed to deny the probability of the fact), are declared by Louis to be identical, anatomically and symptomatically, with the dothinenteritis of Paris: but it is both more fair and more correct to state that there is no shadow of *proof* that true follicular lesion did not exist in numbers of the victims of camp typhus referred to.

II. Dr. Stewart next examines the question of "transmission or propagation from a diseased to a healthy person." The reality of the contagious nature of typhus has, he correctly observes, been generally admitted; but he is in error in questioning the admission of similar, though less marked, contagiousness in the case of dothinenteritis. We have seen that it is now recognized by such French pathologists as have made that malady a subject of serious study; and a certain prejudice, which prevented due investigation of the question, is the only reason of its not having been earlier recognized. Dr. Jackson has seen "four persons employed in the hospital attacked with typhoid fever" (dothinenteritis), within a period of ten days. (Report, p. 34.)

III. "The fact [?] that the mean duration of typhus is about one half that of typhoid fever, is one that perplexes considerably the advocates of their identity." Dr. Stewart states the mean duration of typhus as about 21 days; the inference is that that of dothinenteritis is about 42. That this distinction is far from being universally prevalent appears from the facts very fairly stated by Dr. Stewart himself: in the cases observed by Dr. Shattuck, the mean duration of those with Peyerian lesion was 22½ days, of those without it 24½: the mean duration of 25 Parisian cases of dothinenteritis in 1839-40 (the disease however was less severe than usual,) was no more than 19·6 days. Both these series of cases are far from extensive, however, and therefore let us pass over the evidence they supply. This objection does not apply to 255 cases of recovery from dothinenteritis observed most carefully by Dr. Jackson, whereof the mean duration was precisely 22·019 days. "In the *few instances* where there was a *relapse* into the typhoid fever after a temporary convalescence," this observer "noted the second convalescence as the true one." The words "few instances" are put in italics, because Dr. Stewart finds a new or rather subsidiary motive for distinction in "the frequency of relapse" in dothinenteritis and its rarity in typhus. A passage he quotes from Louis is not capable of the interpretation he would attach to it, and from our own experience of the Parisian fever we can affirm, in opposition to Montault, that *true* relapse is exceedingly uncommon. The cases adduced by Dr. Stewart no doubt prove the fact, but not the frequency of the fact, of a repetition of development of follicular lesion. We might also refer to Dr. Reid's result in respect of duration, which has been already transcribed. But suppose Dr. Stewart had proved to demonstration that continued fever with Peyerian lesion runs a mean course double as long as that of continued fever without such lesion, he would simply have shown the influence of a certain anatomical change in protracting the duration of the malady.

IV. The symptoms referred to by Dr. Stewart are those appertaining to the abdominal viscera and the skin. The reality of marked difference is not denied by any one who has examined a number of patients affected with the two diseases.

V. With reference to the anatomical lesions of the diseases, Dr. Stewart produces no new facts; in respect of the relation of diarrhœa, abdominal pain, and tympanitis to lesion of the patches, he contributes tabular expositions of his own experience confirmatory of results previously obtained, within certain limits, by Dr. Reid. Upon these tables he observes: "What chiefly concerns us to notice is the striking fact that as the number of enlarged follicles increases, the cases of spontaneous diarrhœa diminish, and those in whom consecutive diarrhœa and costiveness were observed become more numerous. In fact, not one of those in whom the greatest number of enlarged follicles was observed but was either constipated, or had diarrhœa brought on by medicine during life. This confirms our former deductions, agrees entirely with those of Valleix, and shows that the appearances observed in typhus depend upon local irritation and not upon specific disease." But to this we reply that at Paris :—1, There is no exact relation between the violence of the abdominal symptoms and the extent of the Peyerian lesion. 2, We have seen cases wherein the merest elevation of the patches saved the diagnosis of dothinenteritis, yet here the abdominal symptoms had not excited attention from their mildness: the disease may be latent altogether, and yet end by the worst form of intestinal lesion—perforation. 3, M. Chomel speaks doubtingly of the relation of the abdominal symptoms to the Parisian lesion. 5, Diarrhœa is stated by Mr. Goodsir to be a rare attendant on the continued fever of Anstruther; yet here, as we have seen, the special lesion of the patches exists with all its anatomical peculiarities; Dr. Anderson, too, observed diarrhœa in five only of twelve cases, of what he terms "pure intestinal disease," or dothinenteritis.

VI. "From the treatment of the two diseases we can infer but little," says Dr. Stewart, and he is right; yet straightway forgetting his own principle, he proceeds to draw inferences from certain fancied therapeutical results. We shall not imitate his oversight, but take leave of his essay by stating that its general character exhibits merit of a very high order.

Dr. Anderson is of opinion that Dr. Stewart has perfectly succeeded in "demonstrating that our typhus is different from the typhoid fever;" but he is disposed to take a different view of the matter somewhat differing from Dr. Stewart's. "I do not think," he observes, "typhus distinct from typhoid fever, as typhus is from smallpox for example; that is, each being a *fever*, properly so called, and therefore incapable of coexisting with each other; but I believe them to be like typhus and pneumonia, perfectly heterogeneous, and therefore capable of being implanted one upon another, typhus being a general febrile disease, affecting the whole system and no part in particular; the 'typhoid fever' being not a fever at all, but a purely local malady, frequently accompanied, as other local affections are, by a febrile state, and sometimes uniting with typhus to form a compound disease, partaking of the symptoms of both." According to Dr. Anderson there are three distinct maladies —"pure intestinal disease," typhus with intestinal disease and typhus without intestinal disease.

English writers, who maintain that the continued fevers of France and England are different maladies, will doubtless triumph at the espousal of their notion by M. Louis. It may consequently be as well to anticipate, and possibly to prevent their jubilee, by showing the exact character of the support they receive from that distinguished source. M. Louis regards as matter of demonstration the identity of the continued fever or dothinenteritis of Paris (A), and of camp, gaol, &c. fever (B); and maintains the non-identity of dothinenteritis (A) and the common English continued fever (C): hence, as A and B are similar quantities, and A and C dissimilar, it follows that B and C are dissimilar. On the other hand, the English writers referred to regard it as a point not open to question that camp, gaol, &c. fever (B), and the common continued fever of England (C) are in nature identical; hence it follows, with the aid of M. Louis' position of the identity of A and B, that C and A are identical, while the object of these writers is to prove them dissimilar. The nature of the argument will appear more distinctly, when the propositions of each party are placed in juxtaposition:

| M. Louis. | English Writers. |
| --- | --- |
| A and B are similar. | B and C are *similar*. |
| A and C are *dissimilar*. | A and B are similar. |
| ∴ B and C are *dissimilar*. | ∴ A and C are *similar*. |

If after this our countrymen like to have M. Louis' support, we can only say we wish them joy of their dilemma.

What is the legitimate inference from the facts and arguments passed in review? The more efficiently to answer this query, let us commence by exhibiting in the most convenient form for comparison, the symptoms and lesions occurring on both sides of the channel.

| France. | Great Britain. |
| --- | --- |
| **A. Abdominal Symptoms.** | |
| **1. *Diarrhœa.*** | |
| Of almost constant occurrence, frequently appearing on the first day, sometimes violent. | Of rare occurrence, (23 in 139 cases, Stewart,) variable in time of origin, scarcely ever violent. |
| **2. *Abdominal Pain.*** | |
| Of almost constant occurrence, often on the first day, obtuse, variable in situation and duration. | Not frequent, often on the first day when it does occur, obtuse, variable in situation and duration. |
| **3. *Meteorism.*** | |
| Exists in more than half the cases, (89 in 134, Louis) often considerable, and of long duration. | Occasionally occurs (74 of 463 cases, Stewart,) commonly slight. |

| France. | Great Britain. |
|---|---|
| **4. *Nausea and Vomiting.*** | |
| Of tolerably frequent occurrence, often arising the first day. | Occasionally appear from the first; thus, nausea in 14 out of 18 cases on the first day, (Anderson). |
| **5. *Deglutition.*** | |
| Occasionally obstructed from organic causes, or deficient innervation. | |
| **6. *Tongue.*** | |
| Exhibits itself in all variety of conditions. | |
| **7. *Intestinal Hemorrhage.*** | |
| In 7 out of 147 cases, (Chomel). | Extremely rare. |
| **B. Symptoms appertaining to the Organs of the Senses.** | |
| **8. *Cephalalgia.*** | |
| In 127 of 134 cases; in 113 of 127 on the first day; ordinary duration eight to ten days. | In 150 of 159 cases; on the first day in 92 out of 108; mean duration ten days, (Henderson). |
| **9. *Somnolence.*** | |
| Very frequent. | Of less frequent occurrence. |
| **10. *Delirium.*** | |
| In more than half the cases; mean period of origin twelfth day; perfectly independent of appreciable lesion of the encephalon. | In about a quarter of the cases; mean period of origin about the eleventh day, (Henderson). Perfectly independent of appreciable lesion of encephalon. |
| **11. *Spasmodic Symptoms.*** | |
| In about one fifth of the cases. | Quite as frequent. |
| **12. *Continued pain in the Limbs.*** | |
| Of frequent occurrence, and extremely unimportant. | |
| **13. *Epistaxis.*** | |
| Of tolerably frequent occurrence. | Of rare occurrence. |
| **14. *State of Eyes.*** | |
| Injection of conjunctiva in about half the cases. | Suffusion of eyes extremely frequent; injection of conjunctiva very common. |
| **15. *Tinnitus Aurium.*** | |
| In about one third of the cases, very rarely at the outset. | Frequency not ascertained. Gerhard found it "very frequent" in the "typhus fever" of Philadelphia. |
| **16. *Deafness.*** | |
| In more than half the cases; developed in middle or towards the close of the disease. | Less frequent *probably;* developed in middle or towards the close of the disease. |
| **17. *Cutaneous Eruption.*** | |
| Occurring in 270 of 294 cases; appearing probably from the sixth to the ninth day, but sometimes earlier; consisting of pale pink coloured spots, slightly prominent, usually few in number, and limited to a small extent of the surface; never observed to pass into the petechial state. | Occurring in 108 of 130 cases, (Henderson,) in 2028 of 2852, (Anderson,) appearing most generally from the fifth to the eighth day, but sometimes earlier, consisting of spots varying in depth of redness, scarcely elevated, usually numerous, and diffused over a considerable extent of surface, and capable of passing (Staberoh, Stewart) into a petechial state. |
| **18. *Cutaneous Hemorrhages.*** | |
| Extremely rare. | Petechiæ* common in some epidemics, rare in others. Vibices very rare. |

* Dr. Stewart (loc. cit.) appears to have shown that true primary petechia, such as are not transformations of eruptive spots, are much more rare than has been supposed.

19. *Sudamina.*

| | |
|---|---|
| In 208 out of 262 cases, (various observers.) | Apparently very rare in this country, Dr. Gerhard, however, only says that their appearance "was not so frequent as in the 'typhoid fever' of Paris." |

20. *Erysipelas.*

| | |
|---|---|
| In 9 of 134 cases, (Louis); in 4 of 42 fatal cases, (Chomel). | In 83 of 2352 cases, (Anderson). |

21. *Cutaneous Sloughs.*

| | |
|---|---|
| In 3 of 57 cases of recovery, (Louis). | Proportion not ascertained. |

22. *Rigors.*

| | |
|---|---|
| In 91 of 109 cases; in 45 of 64 on the first day: a return after they have disappeared announces the occurrence of some secondary lesion. | In 63 of 69 cases; in 48 of 69 on the first day, (Anderson): a return after they have disappeared announces the occurrence of some secondary lesion. |

C. PULMONARY SYMPTOMS.

Catarrhal symptoms and physical signs frequent; pneumonia rare.

D. CIRCULATION.

23. *Pulse.*

Variable in all its characters.

24. *State of the Blood.*

Variable.

E. MODE OF INVASION.

| | |
|---|---|
| In 73 of 112 the invasion was sudden, (Chomel). | The invasion of the disease is sudden in above half the cases, (Anderson). |

F. ANATOMICAL CHARACTERS.

| | |
|---|---|
| A special change in the patches of Peyer is an almost unfailing condition of the existance of the disease. | A special lesion of the patches of Peyer is of extremely rare occurrence, as a general proposition; but in almost all, or in actually all the cases examined at certain periods of the year, or in certain situations that lesion is discovered. When it does exist, the phases through which it passes, and the influence it exercises on the mesenteric glands, are the same as in France. |

G. ETIOLOGY.

The return of the evidence in favour of origin in animal effluvia and contagion is the same in both countries; the evidence is stronger, however, for both in respect of the British malady, but this may be and probably is a merely *apparent* difference. Both maladies are most prone to attack subjects recently immigrated into large towns.

I. DURATION.

| | |
|---|---|
| Commonly greater than that of the continued fever of Great Britain. | Commonly less than that of the continued fever of France. |

K. MORTALITY.

| | |
|---|---|
| Extremely variable, from 5 to 36 per cent. | Extremely variable, from 3 to 50 per cent. |

L. IMMUNITY FROM A SECOND, SECURED BY A FIRST ATTACK.

Supported by the same arguments and proved to the same degree in respect of both maladies.

M. TREATMENT.

The disease cannot be cut short at the outset by any mode of treatment: the influence of therapeutic agency on its mortality and duration is still matter for enquiry: the most advantageous method of treatment is unknown.

While then the "typhoid affection" exists without the Peyerian lesion in extremely rare instances in France, that lesion attends a variable proportion of cases of continued fever in this country; and the same group of *general* functional derangements attends both maladies. Here is sufficient to prove that implication of the glands of the ileum is not a necessary element in the constitution of the disease; hence the lesion in question can scarcely be legitimately put forward as furnishing motive for the separation of the two maladies as distinct species. If the symptoms appertaining to particular organs or systems be made the subject of enquiry, the only difference of moment is found in the condition of the intestinal canal and skin: the diarrhœa, abdominal pain, and meteorism of France are scarcely met with among us; of the sudamina so common there, we commonly see little or nothing; the eruptions are different. Now it is remarkable that in this country while the intestinal lesion is usually absent, the cutaneous eruption is extremely general and extensive; whereas in France the iliac disease is almost invariably present, and the efflorescence limited in extent and slight in degree. Considering this fact in union with that of the well-known reciprocity of function of the skin and intestinal canal, it becomes not altogether improbable that the lesions of these systems are supplementary of each other. That the malady is fundamentally a *general* one appears from its phenomena. This is admitted by Louis and Chomel in respect of the continued fever of Paris; the latter does not allow the Peyerian lesion even so high a rank in secondary importance as the pustules of variola, because these are always, or at least very generally, a measure of the intensity of the disease, which that is not. Few or none doubt it among ourselves, though different interpretations of the phrase "general malady" may be espoused. We know of no single fact proving that this general malady is not one and the same in both countries, though for reasons at present unassignable it is in each attended with a different amount and character of organic implication.

That the form of continued fever attended with disease of the ileum should, during life, be commonly distinguishable by its local abdominal symptoms from those not so attended is nothing more than might be anticipated; and that there are cases in which such distinction cannot be established does much towards demonstrating the secondary importance of the disease in question. But to the idea that any of the general symptoms, or the mean age of the subjects in whom it is developed, characterize the intestinal form, we cannot accede; besides, were such idea substantiated it would obviously not prove any fundamental difference in this from other forms. We cannot accede to it, because, as regards symptoms, proof is not forthcoming; and because in respect of age, while on the one hand to draw a final inference from Dr. Shattuck's thirteen cases is certainly unwarrantable, we are not by any means persuaded that the alleged limitation of the "typhoid affection" of Paris to subjects aged under fifty or thereabouts, is satisfactorily established. Not long since it was matter of faith in Paris that the disease did not occur in infancy, (an opinion which M. Louis has been, without any particle of reason, charged with promulgating); the question having been properly examined, it is now known to be extremely frequent among children: is it not fair to anticipate the occurrence of a similar change of opinion respecting persons of advanced age, at least to acknowledge its possibility?

We believe, then, that the continued fevers of both countries are the

*same species* of disease; but they are *different varieties* of that species. And there is nothing in the climate of either country, or in the influences, external and otherwise, to which their populations are exposed, capable of preventing the evolution of that form of the malady more usual in the other: the French variety is observed, though rarely, in Great Britain; the British, with similar restriction, in France. Some questions of importance remain to be decided: for example, wherein lies the cause of the almost invariable establishment of intestinal special lesion in France, and of its ordinary absence here; whence comes it that at Edinburgh the proportion of fatal cases, with lesion of the patches, amounts during a series of years to but a small fraction of the whole, while at thirty miles from that town no case is examined without their being discovered; why does the proportional number of cases with Peyerian lesion vary in the same locality with different years?

Finally, in adopting the opinion that continued fever is a *general malady*, we do not lend ourselves to the notion of its "*essentiality*," a notion to us just as incomprehensible as that there should be an effect without a cause. We believe, as all sound argument goes to show, that the *blood* is the constituent of the body, implicated from the first; though from the imperfect state of knowledge concerning the normal condition of that fluid, as well as from the imperfection of our modes of estimating its aberrations therefrom, the power of physically demonstrating this is at present and may long continue to be withheld. Meanwhile we think the judicious pathologist can hardly doubt that it is wisdom to adopt the creed, of which, while rational views militate in its favour, no fundamental principle of logic proclaims the fallacy.

---

### Art. II.

*Guy's Hospital Reports.* Nos. X-XI-XII. *April and October*, 1840, *and April*, 1841.—*London.* 8vo.

